# Recombinant Oncolytic Vesicular Stomatitis Virus Expressing Mouse Interleukin-12 and Granulocyte-Macrophage Colony-Stimulating Factor (rVSV-dM51-mIL12-mGMCSF) for Immunotherapy of Lung Carcinoma

**DOI:** 10.3390/ijms26178567

**Published:** 2025-09-03

**Authors:** Anastasia Ryapolova, Margarita Zinovieva, Kristina Vorona, Bogdan Krapivin, Vasiliy Moroz, Nizami Gasanov, Ilnaz Imatdinov, Almaz Imatdinov, Roman Ivanov, Alexander Karabelsky, Ekaterina Minskaia

**Affiliations:** 1 Department of Gene Therapy, Sirius University of Science and Technology, 1 Olympic Avenue, 354340 Sochi, Russia; 2 Federal Budgetary Research Institution State Research Center of Virology and Biotechnology “Vector”, 630559 Novosibirsk, Russia

**Keywords:** oncolytic viruses (OVs), vesicular stomatitis virus (VSV), anti-cancer therapy, oncology, lung carcinoma, immunotherapy

## Abstract

The unique ability of oncolytic viruses (OVs) to replicate in and destroy malignant cells while leaving healthy cells intact and activating the host immune response makes them powerful targeted anti-cancer therapeutic agents. Vesicular stomatitis virus (VSV) only causes mild and asymptomatic infection, lacks pre-existing immunity, can be genetically engineered for enhanced efficiency and improved safety, and has a broad cell tropism. VSV can facilitate targeted delivery of immunostimulatory cytokines for an enhanced immune response against cancer cells, thus decreasing the possible toxicity frequently observed as a result of systemic delivery. In this study, the oncolytic potency of the two rVSV versions, rVSV-dM51-GFP, delivering green fluorescent protein (GFP), and rVSV-dM51-mIL12-mGMCSF, delivering mouse interleukin-12 (mIL-12) and granulocyte-macrophage colony-stimulating factor (mGMCSF), was compared on the four murine cancer cell lines of different origin and healthy mesenchymal stem cells (MSCs) at 24 h post-infection by flow cytometry. Lewis lung carcinoma (LL/2) cells were demonstrated to be more susceptible to the lytic effects of both rVSV versions compared to melanoma (B16-F10) cells. Detection of expression levels of antiviral and pro-apoptotic genes in response to the rVSV-dM51-GFP infection by quantitative PCR (qPCR) showed lower levels of *IFIT*, *RIG-I*, and *N-cadherin* and higher levels of *IFNβ* and *p53* in LL/2 cells. Subsequently, C57BL/6 mice, infused subcutaneously with the LL/2 cells, were injected intratumorally with the rVSV-dM51-mIL12-mGMCSF 7 days later to assess the synergistic effect of rVSV and immunostimulatory factors. The in vivo study demonstrated that treatment with two rVSV-dM51-mIL12-mGMCSF doses 3 days apart resulted in a tumor growth inhibition index (TGII) of over 50%.

## 1. Introduction

Lung cancer accounts for about 13% of all cancers and is the deadliest type of cancer worldwide, leading to more deaths than colorectal, breast, brain, and prostate cancers [1]. Current therapeutic approaches have improved patients’ survival; however, further efforts are required to increase effectiveness and protection against cancer relapse and metastasis.

OVs can be used as part of the immunotherapeutic approach, as they can selectively infect tumor cells, triggering an immune response both against the viral infection and against the tumor cells [2]. Genetic modification of viral genomes can further increase OV tropism for cancer cells, reduce tropism for healthy cells, and increase anti-tumor immune response as a result of OV-mediated delivery of cytotoxic, immunomodulatory, or pro-apoptotic genes [3,4].

Various versions of the VSV have been investigated for their oncolytic potential. VSV, a non-segmented negative-strand RNA virus, belongs to the family *Rhabdoviridae*, and its rapid replication cycle and wide host cell range make it a promising therapeutic agent [5]. Tumor selectivity of VSV is predominantly based on defects in the antiviral defense capabilities of many malignant cells that express only small amounts of type I interferons (IFNs) and/or have defective signal transduction [6]. Additional advantageous VSV features include the ability to replicate in the hypoxic tumor microenvironment (TME) [7,8] and induce tumor cell pyroptosis via activation of the caspase-3/gasdermin E (GSDME) axis with an anti-tumor response [9,10]. The oncolytic properties of recombinant VSVs (rVSV), mainly delivering IFNβ, are studied in clinical trials (NCT01628640, NCT05644509, NCT03865212, NCT06508463, NCT02923466, NCT03017820, NCT03120624, and NCT03647163, www.clinicaltrials.gov, accessed on 20 May 2025). Nevertheless, it is important to remember that several studies demonstrated the insufficient selectivity and strong neurotoxicity of the wild-type (wt) VSV, which led to the development of several improved modified versions, such as the one containing a dM51 deletion, which affects its ability to inhibit the antiviral response (specifically type I IFN) in healthy cells [6]. Another crucial point to remember is the variable sensitivity levels of different cancer cell lines to the VSV oncolytic effect, which may reflect heterogeneity of clinical response to virotherapy. In this study, we compared the susceptibility of murine cancer cell lines of different origin—melanoma (B16-F10), Lewis lung carcinoma (LL/2), squamous-cell carcinoma (SCC VII), and hepatocellular carcinoma (H22)—to the oncolytic effects of rVSV-dM51-GFP and rVSV-dM51-mIL12-mGMCSF. qPCR analysis was performed to analyze the differences in expression levels of certain genes, such as antiviral and pro-apoptotic, in cancer cell lines that are less (B16-F10) or more (LL/2) susceptible to the rVSV-dM51-GFP infection. The therapeutic effect of rVSV-dM51-mIL12-mGMCSF on Lewis lung carcinoma was also assessed in vivo in C57BL/6 mice.

## 2. Results

### 2.1. Production and Characterization of rVSVs

The replication-competent rVSV-wtM-GFP, rVSV-dM51-GFP, and rVSV-dM51-mIL12-mGMCSF were produced as previously described via the helper virus-free method [11]. HEK293TN cells were co-transfected with the plasmids expressing the four VSV proteins (phosphoprotein P, glycoprotein G, nucleocapsid N, and matrix protein M) under the control of the cytomegalovirus immediate-early enhancer/chicken β-actin promoter (CAG), pCAG-T7pol, and one of the “core plasmids”: pVSV-wtM-GFP, pVSV-dM51-GFP (transgene size 700 bp), or pVSV-dM51-mIL12-mGMCSF (transgene size 2052 bp). GFP expression and cytopathic effect (CPE) were observed 24 h post-transfection and 72 h after supernatant infection, respectively. The amplification of rVSVs in BHK-21 cells involved infection with post-transfection-conditioned culture media containing virus particles and three rounds of virus amplification. The virus titer was assessed by CPE in BHK-21 cells 72 h post-infection. The concentration of rVSV preparation was 3 × 10^9^ TCID50/mL.

For the in vivo experiment, the purification of rVSV-dM51-mIL12-mGMCSF was carried out by ultracentrifugation with a sucrose cushion. The viral preparation was diluted to a final concentration of 1 × 10^7^ TCID50 in a 50 µL volume.

To confirm the activity of mGMCSF present in rVSV-dM51-mIL12-mGMCSF supernatants, the mGMCSF ELISA Kit (EM0089, FineTest, Wuhan, China) was used. The optical density (OD450) values of the samples containing rVSV-dM51-mIL12-mGMCSF supernatants from BHK-21 cells were statistically significantly higher relative to the negative control (NC) than those of the samples containing rVSV-dM51-GFP (Figure S1). We have also previously demonstrated the activity of mIL-12 on a HEK-Blue™ IL-12 reporter line [11].

### 2.2. Lewis Lung Carcinoma Cells LL/2 Are More Susceptible to the VSV Oncolytic Effect than the B16-F10 Melanoma Cells

**Figure 1:**
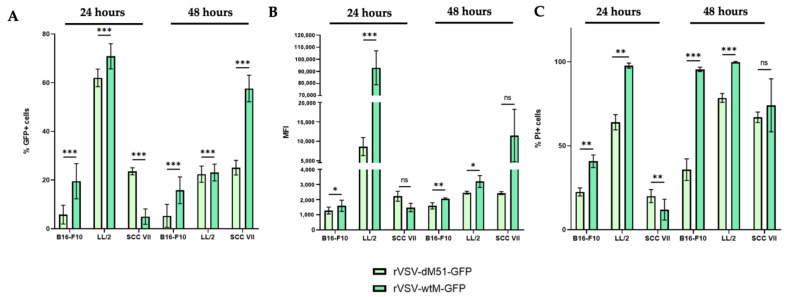
Flow cytometry analysis of B16-F10, LL/2, and SCC VII cells at 24 and 48 h post-infection with rVSV-dM51-GFP and rVSV-wtM-GFP. The number of GFP+ cells (**A**), MFI values (**B**) and PI+ cells (**C**) (* *p*-value ˂ 0.05, ** *p*-value ˂ 0.01, *** *p*-value ˂ 0.001, not significant (ns)—*p*-value > 0.05).

Methionine at position 51 of the M protein was previously demonstrated to be crucial for the inhibition of the IFN type I- and NFKB1-mediated immune response [12]. rVSV-dM51-GFP has a deletion of this residue compared to rVSV-wtM-GFP, which prevents rVSV-dM51-GFP from binding to the Rae1-Nup98 mRNA export complex necessary for cellular mRNA transport and subsequent translation [13]. The inability to suppress the antiviral response in VSV-infected cells (healthy or cancerous) by disruption of the transport and translation of the cellular mRNAs coding for antiviral genes leads to the limited distribution of rVSV-dM51-GFP [14]. The initial aim of this study was to compare the replicative activity of the two rVSV versions (rVSV-wtM-GFP and rVSV-dM51-GFP) in three murine cancer cell lines: B16-F10, LL/2, and SCC VII. Our previously published data [11] demonstrated that the B16-F10 cells were not highly susceptible to the VSV oncolysis in vivo. The aim of this study was to understand the reason behind this finding through assessment of differences between cancer cell lines with different sensitivity to OV that may provide a clue for the development of efficient virotherapy. As expected, rVSV-wtM-GFP demonstrated higher potency as compared to rVSV-dM51-GFP at 24 h post-infection, shown by the number of GFP+ cells, MFI values, and the number of PI+ cells, which were 3.4, 1.3, and 1.8 times higher in B16-F10 and 1.1, 10.8, and 1.5 times higher in LL/2 cells, respectively (Figure 1 and Table S1). Infection of B16-F10 cells with rVSV-dM51-GFP resulted in 5.8% GFP+ (MFI 1279) and 22.5% PI+ cells, while infection of LL/2 cells resulted in 62.0% GFP+ (MFI 8635) and 64.0% PI+ cells. Infection of B16-F10 cells with rVSV-wtM-GFP resulted in 19.5% GFP+ (MFI 1593) and 40.8% PI+ cells, while infection of LL/2 cells resulted in 70.9% GFP+ (MFI 93004) and 97.8% PI+ cells. The number of LL/2 PI+ cells was 2.8 (rVSV-dM51-GFP) and 2.4 (rVSV-wtM-GFP) times higher as compared to B16-F10 cells. Interestingly, the opposite trend was observed in SCC VII cells: rVSV-dM51-GFP was more potent than rVSV-wtM-GFP during the first 24 h post-infection. Why this particular cell type is initially more permissive to rVSV-dM51-GFP but not the wild-type virus replication is extremely interesting and remains to be investigated in the future.

The observed decreasing numbers of GFP+ cells and the lower MFI values at 48 h post-infection were due to most cells being lysed by the rVSVs and turning into cellular debris. Interestingly, by this time point the trend in SCC VII cells reversed, and the rVSV-wtM-GFP infection resulted in 57.6% GFP+ cells (MFI 11455), which was 2.3 (4.7) times higher as compared to rVSV-dM51-GFP. Statistical analysis of the obtained data (*t*-test) confirmed that the observed differences between rVSV-dM51-GFP and rVSV-wtM-GFP were statistically significant.

**Figure 2:**
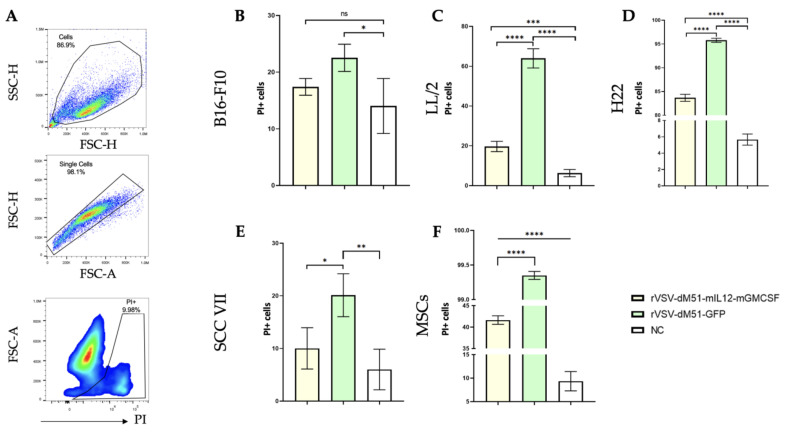
Flow cytometry analysis of the four murine cancer cell lines and healthy MSCs at 24 h post-infection with rVSV-dM51-GFP and rVSV-dM51-mIL12-mGMCSF. (**A**) Gating strategy. The statistical analysis of the number of PI+ (dead) B16-F10 (**B**), LL/2 (**C**), H22 (**D**), SCC VII (**E**), and MSC (**F**) cells. (*) *p*-value < 0.05, (**) *p*-value < 0.01, (***) *p*-value < 0.001, (****) *p*-value < 0.0001, not significant (ns)—*p*-value > 0.05.

Next, we compared the oncolytic effects of the two rVSV-dM51 versions, rVSV-dM51-GFP and rVSV-dM51-mIL12-mGMCSF, at 10^6^ TCID50 on the extended cell panel: B16-F10, LL2, SCC VII, and H22 cancer cell lines and healthy bone marrow MSCs. Infection with these viruses caused CPE of various degrees in all cell lines, and the number of dead cells was assessed by flow cytometry at 24 h post-infection. Uninfected live single cells (Figure 2A) were used as a negative control (NC) for setting the gate for the PI+ population. The rVSV-dM51-mIL12-mGMCSF infection of B16-F10, LL2, SCC VII, and H22 cells resulted in 17, 22, 7, and 84% PI+ cells, which was 1.3 (23% PI+), 3.2 (64% PI+), 2.0 (20% PI+), and 1.1 (96% PI+) times lower, respectively, as compared to the number of PI+ cells as a result of rVSV-dM51-GFP infection (Figure 2B and Table S2).

These results further demonstrate that the B16-F10 melanoma cell line is significantly less susceptible to the oncolytic effect of rVSVs than the LL/2 lung carcinoma and H22 hepatocellular carcinoma cell lines and that the insertion of the additional longer sequence encoding mIL12-mGMCSF (2052 bp) decreases the oncolytic potency in vitro as compared to rVSV-dM51-GFP, delivering GFP (700 bp). This is, unfortunately, in accordance with the previously published [15] data, which demonstrated that viral oncolytic activity is reduced by the insertion of bigger genes. Importantly, statistical analysis demonstrated high statistical significance (*p*-value < 0.0001) between rVSV-dM51-GFP and rVSV-dM51-mIL12-mGMCSF in the four cancer cell lines.

The TME is a complex system consisting of cancerous and healthy cells: immune cells, endothelial cells, fibroblasts, MSCs, and other components that closely interact with each other to support tumor growth [16,17,18]. TME (in particular, stromal cells and MSCs) can stimulate cancer cell proliferation, suppress apoptosis, and promote angiogenesis and metastasis. At the same time, non-cancerous cells should be able to activate the potent response against OVs. With that notion in mind, we wanted to assess the ability of the rVSV to infect and lyse healthy murine MSCs. As demonstrated, rVSVs actively replicated in MSCs, leading to their effective oncolysis: more than 40% and nearly 100% of rVSV-dM51-mIL12-mGMCSF- and rVSV-dM51-GFP-infected cells, respectively, were PI+ (Figure 2F and Table S3). Statistically significant differences were demonstrated both between rVSV-dM51-GFP- and rVSV-dM51-mIL12-mGMCSF-infected MSCs and between the infected cells and the NC (*p*-value < 0.0001). This is an interesting finding, which potentially may help counteract the negative effects of the TME MSCs and is further supported by the published research that demonstrated that healthy MSCs, just like malignant cells, have defects in their type I IFN response, which explains their susceptibility to the OV-mediated lysis [19,20].

### 2.3. Induction of Antiviral and Pro-Apoptotic Genes in Response to rVSV Infection in B16-F10 and LL/2 Cells

The level of susceptibility to the virus-mediated oncolysis depends on the intrinsic abilities of particular cancer cell types to fight off the viral infection. Therefore, it was important to explore the differences in the expression levels of certain antiviral (such as *IFN-β1*, *IFIT1*, and *RIG-I*), pro-apoptotic (such as *p53*), and metastatic (such as *N-cadherin*) genes in the two cancer cell lines: the less susceptible to rVSV oncolysis B16-F10 and the very sensitive to this action LL/2.

**Figure 3:**
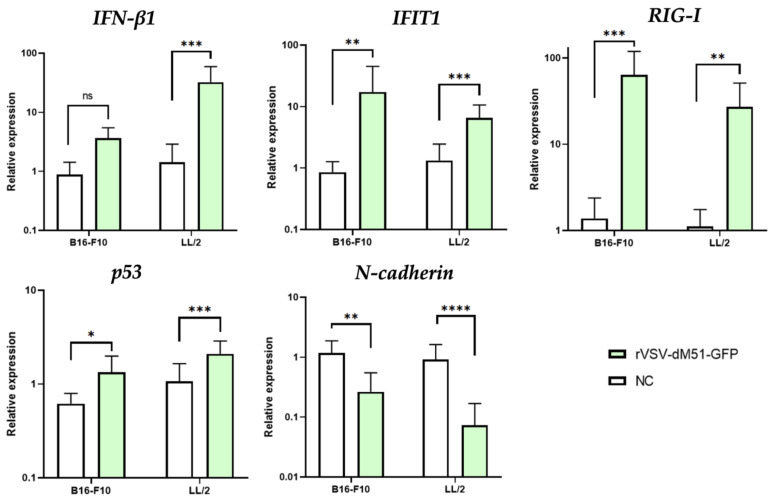
Induction of gene expression in response to VSV infection. qPCR analysis of the rVSV-dM51-GFP–infected B16-F10 and LL/2 cells at 12 h post-infection. (*) *p*-value < 0.05, (**) *p*-value < 0.01, (***) *p*-value < 0.001, (****) *p*-value < 0.0001, not significant (ns)—*p*-value > 0.05.

The sensitivity of cancer cells to VSV and OVs in general (compared to the healthy cells) is mainly attributed to the low expression levels of type I IFNs (IFNα and IFNβ) and overall to the various defects in receptors and the active members of type I IFN signaling. The assessment of expression levels of *IFN-β1* by qPCR 12 h after rVSV-dM51-GFP infection demonstrated that *IFN-β1* expression in LL/2 cells was 9 times higher as compared to B16-F10 cells (Figure 3). A similar trend in expression was observed for the pro-apoptotic *p53*—its level of expression in LL/2 was 1.6 times higher as compared to B16-F10 cells (Figure 3). The expression levels of the two antiviral genes (*IFIT1* and *RIG-I*) and the metastatic *N-cadherin* were 2.6, 2.3, and 4.3 times higher, respectively, in B16-F10 cells as compared to LL/2 (Figure 3). The data on the relative expression levels of *IFIT1* and *RIG-I* demonstrates the strength of the early antiviral response by the B16-F10, which is less sensitive to VSV oncolysis. However, it is important to note that the presence of mRNA transcripts demonstrates successful transcription yet does not equal successful protein translation. Importantly, the changes in relative expression levels of the five studied genes in response to the viral infection were (with the exception of *IFN-β1* in B16-F10 cells) statistically significant as compared to the NC.

### 2.4. Intratumoral Injection of rVSV-dM51-mIL12-mGMCSF Suppresses the LL/2-Induced Tumor Growth

**Figure 4:**
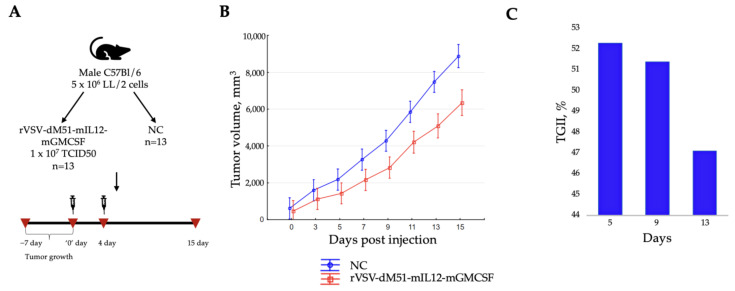
rVSV-dM51-mIL12-mGMCSF inhibits LL/2-induced tumor growth. (**A**) Study design. (**B**) Tumor growth measured in mice for 15 days post-first rVSV-dM51-mIL12-mGMCSF treatment. Statistically significant differences in tumor volumes between treated (rVSV-dM51-mIL12-mGMCSF) and NC groups were observed during 12 days of the study (days 3 to 15) (Mann–Whitney U-test, *p*-value < 0.05). Partially inhibited tumor growth in treated group mice reached a maximum of 52.3% on day 5. (**C**) TGII in rVSV-dM51-mIL12-mGMCSF-trated mice on days 5, 9 and 13.

The data obtained in vitro demonstrated that the B16-F10 melanoma cell line is significantly less susceptible to the oncolytic effect of rVSVs than the LL/2 lung carcinoma and H22 hepatocellular carcinoma cell lines. Due to the observation that the H22-induced tumors spontaneously regressed in BALB/c mice, and in keeping with the same mouse model, the more susceptible to rVSV oncolysis LL/2 cells were used in the in vivo experiment as the more optimal model than the B16-F10 that previously demonstrated a 37% TGII [11]. Therefore, C57BL/6 mice were injected subcutaneously with 5 × 10^6^ LL/2 cells, and the tumor sizes were measured every 2 days for 15 days (Figure 4A). The treatment with rVSV-dM51-mIL12-mGMCSF commenced on day 7 post-infusion, when the average tumor size reached ~400 mm^3^. Two rVSV-dM51-mIL12-mGMCSF injections 3 days apart were delivered intratumorally to the treatment group (1 × 10^7^ TCID50) mice. NC mice received phosphate-buffered saline (PBS) only. Overall, rapid tumor growth was observed in all groups due to the nature of the LL/2-induced carcinoma, but this process in the placebo group was faster (Figure 4B). Partially inhibited tumor growth was observed in treated mice compared to the NC group during 15 days of observation, with TGII reaching its maximum of 52.3% on day 5 (Figure 4C). Statistically significant differences in tumor volumes between the two groups were observed for 12 days (days 3 to 15) of the study (Mann–Whitney U-test, *p*-value < 0.05). The differences in survival between the two groups were statistically insignificant.

**Figure 5:**
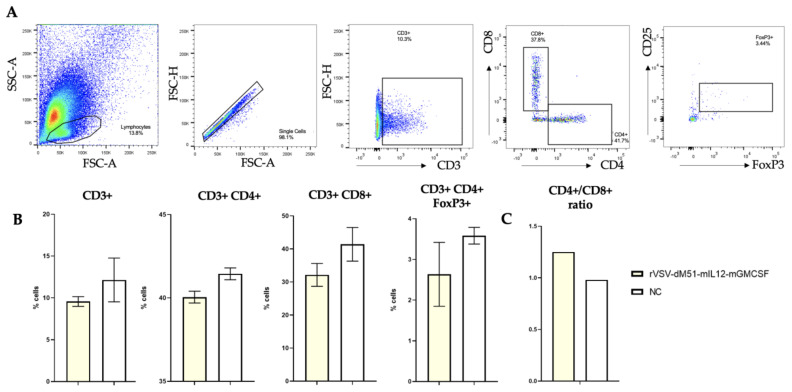
Flow cytometry analysis of the immunophenotypic profile of rVSV-dM51-mIL12-mGMCSF-treated and NC mouse spleen lymphocytes. (**A**) Gating strategy. (**B**) Percentage of cells within each immune cell population. (**C**) CD4^+^/CD8^+^ NC to treated ratio.

The immune phenotypic profile of the treated and NC mouse spleen lymphocytes, isolated on the last day of the experiment, was studied by flow cytometry with anti-CD3, CD4, CD8, CD25, and FoxP3 antibodies. The following immune cell populations were analyzed: total CD3^+^, CD3^+^ CD4^+^ helper T lymphocytes, CD3^+^ CD8^+^ cytotoxic T lymphocytes, and CD4^+^ CD25^+^ Foxp3^+^ regulatory T lymphocytes (Treg) (the gating strategy is shown in Figure 5A), as well as the CD4^+^/CD8^+^ ratio. The numbers of CD3^+^, CD3^+^ CD4^+^, CD3^+^ CD8^+^, and CD4^+^ CD25^+^ Foxp3^+^ T cells were overall higher in the NC group, and no statistically significant differences were observed between the immune cell populations from the rVSV-dM51-mIL12-mGMCSF-treated spleens and the NC using Student’s *t*-test (Figure 5B). However, the CD4^+^/CD8^+^ ratio was increased (1.25) and 1.3 times higher in the VSV treatment group as compared to the NC (0.98) (Figure 5C). However, considering the limited sample size and the absence of statistically significant differences, while the data is valuable, further studies with higher numbers of samples and cells would need to be conducted to make firm conclusions.

## 3. Discussion

OV-based therapy is a promising approach for the treatment of oncological diseases, especially those resistant to conventional therapies. Since the discovery of oncolytic qualities in some viruses, various strategies for their improvement have been employed with the aim of increasing the oncoselectivity and enhancing the suppressed immune response of the host against cancer. Due to the wild-type VSV’s insufficient selectivity and strong neurotoxicity, its oncoselectivity and safety can be improved by introducing various modifications [4]. Several approaches have been used so far to create a safe and effective VSV-based therapy: introduction of mutations into the G protein to limit/direct VSV tropism or virus pseudotyping for inhibition of VSV neurotropism; VSV attenuation via disruption of the normal gene order; encoding expression of various immunostimulatory genes encoded in the VSV genome; and introduction of microRNA targets into the VSV genome for inhibition of VSV-associated toxicities [21]. It is, however, worth mentioning that the insertion of additional sequences coding for non-VSV genes (such as GFP or IL12-GMCSF) tends to decrease virus potency in reverse proportion to the insert size [11,15]. Prior in vitro screening of rVSV on various cancer cell types is extremely important for the future success of preclinical studies since susceptibility to VSV-mediated lysis differs depending on cancer type. There are many differences between cell lines for each type of cancer. These differences are not only cancer type-specific but also patient-specific, and these differences often define the success (and, unfortunately, lack thereof) of therapies. Therefore, it would be incorrect (based only on one lung cancer cell line) to make sweeping generalizations and firm conclusions that rVSV is most potent on lung cancer (any lung cancer, for that matter). Based on the results presented in this study, the most sensitive of the tested cell lines, Lewis lung carcinoma, was chosen for the subsequent in vivo experiment. With that in mind, it would be interesting, in future studies, to analyze the sensitivity of various lung adenocarcinoma lines to the rVSV in order to get a deeper understanding and prediction of its future therapeutic efficacy on the specific lung cancer types. However, the murine Lewis lung carcinoma model is also considered a valuable preclinical model for human lung carcinoma. This gives hope that rVSV will be just as potent in the future clinical studies.

Ideally, the data on assessment of viral lytic ability should be additionally enriched by the data on induction of the signaling cascades in the infected cells. We previously demonstrated that the B16-F10 cell line was not highly sensitive to VSV-mediated oncolysis. Therefore, we first compared the ability of the two rVSV versions (wtM and dM51) to replicate in and lyse three tumor cell lines: melanoma, Lewis lung carcinoma, and squamous-cell carcinoma. Predictably, rVSV-wtM-GFP demonstrated a significantly higher oncolytic effect in all three cell lines compared to rVSV-dM51-GFP, and LL/2 cells were much more susceptible to VSV oncolysis as compared to B16-F10 cells. Indeed, the use of the rVSV-wtM-GFP, in terms of its potency, would be much more effective, and our in vitro data additionally supports this notion. However, the introduction of the dM51 is important, as VSV versions with mutations in the M protein which are considered safer as therapeutic agents dominate the clinical studies.

The present study also showed that healthy MSCs were even more susceptible to rVSV oncolysis than the LL/2 cells. This is an interesting and important finding, as TME MSCs were shown to stimulate cancer cell proliferation, suppress apoptosis, and promote metastasis, and their apparent susceptibility to VSV-mediated oncolysis as a result of the defects in their type I IFN response [6] may help counteract their negative effects on tumor progression. Not unexpectedly and unfortunately, the insertion of a bigger transgene (IL12-GMCSF) in place of GFP decreased viral potency in vitro. Interestingly, a similar phenomenon of the bigger load reducing oncolytic efficiency was observed in the study comparing recombinant vaccinia viruses delivering either red fluorescent protein (RFP) or bacterial flagellin (subunit B) of *Vibrio vulnificus* (FlaB) fused to RFP [15].

Successful viral entry depends on the receptor repertoire. According to published studies, the low-density lipoprotein receptor (LDLR) serves as the major entry point for VSV, and the widespread expression of LDLR family members accounts for the VSV pantropism [22]. Several lines of evidence have supported a link between LDLR and carcinomas, demonstrating the increase in LDLR activity during the growth phase and decrease in quiescent cells in a number of carcinoma cell lines [23,24] and tumor growth suppression in LDLR-deficient mice [25]. VSV, like some other enveloped viruses, also interacts with the epidermal growth factor receptor (EGFR) to facilitate viral entry and replication and stimulates the EGFR/AKT endocytosis signaling pathways [26,27]. EGFR is a highly glycosylated and phosphorylated transmembrane receptor tyrosine kinase (RTK) that regulates cell proliferation, survival, tumorigenesis, and type I IFN signaling [28]. EGFR overexpression is correlated with the risk of metastasis, resistance to chemotherapy, and poor survival for patients with breast cancer, some types of melanoma, head and neck squamous cell carcinoma, and non-small cell lung carcinoma [29,30,31]. Viral entry increases FUT8 expression, thus promoting viral RNA replication via suppression of the type I IFN response through core fucosylated-EGFR-JAK1-STAT3-RIG-I signaling. Kinase inhibitors, such as gefitinib, affecting EGFR and STAT1/2 phosphorylation as well as NFKB signaling, negate the effects of vanadium-based compounds, which sensitize cells to the OV oncolysis and not only attenuate the type I IFN antiviral response but also increase type II IFN-mediated pro-inflammatory gene induction when co-administered with rVSV-dM51, as demonstrated by the increased phosphorylation of EGFR and the downstream extracellular signal-regulated kinase 1/2 (ERK1/2) [32,33]. At the same time, VSV M protein either inhibits the nuclear export of mRNAs [34,35] or interferes with JAK/ STAT signaling [36]. Therefore, while the wild-type VSV triggers a primary antiviral response, the secondary response is suppressed by blocking the export of critical antiviral mRNAs, such as IFN-β1 mRNA, from the nucleus to the cytoplasm; the attenuated rVSV-dM51 with modified M does not possess the IFN-suppressive qualities [32]. As was previously demonstrated by Lai et al. [37], the level of EGFR expression in LL/2 lung carcinoma cells is significantly higher than that in melanoma B16-F10, which could explain their susceptibility to VSV-based oncolysis. Several studies took advantage of EGFR upregulation in certain malignancies for increased therapeutic specificity of OVs [29,38,39] and there is evidence suggesting that activation of the EGFR pathway bypasses the IFN-regulated antiviral response, leading to viral replication [40,41,42].

The qPCR analysis of mRNA transcripts of several genes in response to VSV infection of melanoma and Lewis lung carcinoma cell lines in vitro could potentially provide the answer to the observed differences in viral oncolysis. The antiviral response involves detection of viral RNA by RIG-I and induction of a signaling cascade to produce IFNβ, which then induces the expression of IFIT. Inhibition of the viral protein translation by IFIT prevents viral replication. Our qPCR data demonstrated that the expression levels of *IFNβ* and *p53* were lower in B16-F10 cells, while those of *IFIT*, *RIG-I*, and *N-cadherin* were higher compared to LL/2 cells. A situation with low *IFNβ* but higher *IFIT* and *RIG-I* levels observed in B16-F10 cells points to these cells trying to evade the antiviral response by interfering with downstream signaling, despite the initial RIG-I recognition of the danger presented by VSV. The high *IFIT* expression indicates that the downstream IFNβ-induced genes may be active, but the significantly lower *IFNβ* expression points to a dysfunction in the signaling cascade after RIG-I activation but before IFNβ production by the melanoma cells. It is also possible that the antiviral response is incomplete or dysregulated. In LL/2 cells, on the other hand, the presence of high levels of *IFNβ* (9 times higher than those in B16-F10 cells), but low *RIG-I* and *IFIT*, points to a suppressed or dysfunctional antiviral response and, hence, a reduced ability to block the virus from multiplying. While high *IFNβ* levels, not driven by RIG-I activation and likely sustained by other mechanisms, are produced, the lower levels of *RIG-I* and *IFIT* indicate that LL/2 cells do not detect the OV or respond to it effectively. In addition to higher *IFIT* and *RIG-I* levels, the level of *N-cadherin* mRNA transcripts was over 4 times higher in melanoma, which may point to promotion of metastasis by IFIT, in which case development of IFIT-inhibiting drugs might improve the outcomes. It would be very interesting and important to further study and correlate the observed differences in the antiviral genes’ levels with the sensitivity of various cancer types (and in particular, lung cancer lines) to virotherapy, which may, in the future, pave the way to the development of drugs sensitizing cancer cells that are resistant to OVs and to improve the outcomes of virotherapy.

With that in mind, it is important to highlight several factors that may affect the interpretation of the obtained results: the selected time point (12 h), the fact that the mRNA levels often do not directly correlate with protein levels and activity (especially as mentioned previously for cancer cells and type I IFN signaling members), and/or the in vitro conditions of the experiment, which do not reliably reflect the interaction of the virus with cancerous cells and the immune cells within TME during the in vivo treatment. In order to draw plausible conclusions from the qPCR data, it would be important to conduct thorough research and carry out Western blotting and flow cytometry analysis in the future.

Previously, our in vivo studies demonstrated that the mIL12-mGMCSF fusion protein delivery by rVSV-dM51 increased the therapy efficiency (despite its decreased potency in vitro) compared to the virus without the immunostimulatory payload and resulted in significantly reduced tumor volumes and a prolonged life span of the mice with B16-F10 melanoma [11]. Published studies showed that both IL-12 and GM-CSF stimulate potent anti-tumor responses: IL-12 activates IFN-γ production in NK, CD4^+^, and CD8^+^ T lymphocytes and induces T-helper 1 (Th1) differentiation [43,44,45,46], while GM-CSF promotes the maturation and activation of cross-presenting dendritic cells (DCs) [47]. For example, inhibition of xenograft growth and glioblastoma metastases was achieved as a result of intratumoral injection of the vaccinia virus delivering GM-CSF and the oncotoxic protein lactaptin [48]. Notably, the first OV-based therapy approved for the treatment of melanoma in the USA in 2015 delivered the human GM-CSF for enhanced immune response, so this immune stimulator proved itself as a reliable therapeutic, albeit not 100% effective.

Our pilot in vivo experiment assessing the effect of rVSV-dM51-mIL12-mGMCSF on LL/2-induced tumor growth demonstrated significantly enhanced tumor growth inhibition (52.3%) on day 5 in a group of animals that received two doses of OV therapy as compared to the NC. This effect lasted for 15 days post-rVSV treatment. Inhibition of LL/2-induced tumors by this rVSV was more significant (15% higher) compared to our data obtained previously on the B16-F10 melanoma model (about 37%) [11]. Interestingly, while no statistically significant differences in CD4^+^ and CD8^+^ numbers between the treated and NC group spleens were observed, the CD4^+^/CD8^+^ T cell ratio in the treatment group (1.25) was higher than in the NC (0.98). The CD4^+^/CD8^+^ ratio can be used as a biomarker predicting cancer treatment response and prognosis. An elevated CD4^+^/CD8^+^ ratio in treatment-responsive tumors suggests a better response to a therapy. The lower (albeit not statistically significant) numbers of Treg cells observed in the treated group spleens compared to the NC is a rather positive finding since the presence of higher Treg numbers in TME is a negative cancer prognostic factor demonstrating the suppression of anti-cancer immune cell activity. However, the small number of studied samples and cells and the lack of diverse time points and statistical significance present a limitation that prevents drawing firm conclusions.

Clearly, delivery of immunostimulatory factors in vitro does not affect the sensitivity and gene expression profile of cancer cell lines. Additional in vivo experiments are necessary to refine, support, and expand the obtained data. Comparison of rVSV-dM51-mIL12-mGMCSF and rVSV-dM51-GFP in vivo will allow researchers to assess the effect of virotherapy alone and the additional delivery of immunostimulatory factors, as well as to study the phenotype of TME cells (such as MSCs, M2 macrophages, and dendritic cells) rather than splenocytes, which will be more informative. Unfortunately, the significant time and resources required to carry out the additional experiments (testing the sensitivity of the various lung cancer cells to rVSV oncolysis, analyzing the gene expression profile in response to rVSV infection, in vivo experiments, and phenotyping TME cells at various time points) mean it will not be possible to immediately get all the answers to the sought questions. Therefore, these experiments will have to become the focus of future study.

Taken together, our findings indicate that Lewis lung carcinoma was a more optimal model as compared to melanoma for rVSV virotherapy. More cancer cell lines would need to be studied to confirm that the conclusion drawn based on our data is applicable to all types of lung cancers (and not only of mouse but, more importantly, human origin). Further in-depth study will help to decipher the intrinsic processes driving the anti-tumoral activity of rVSV-dM51-mIL12-mGMCSF.

## 4. Materials and Methods

### 4.1. Cell Lines

Baby hamster kidney fibroblasts (BHK-21), human embryonic kidney 293 (HEK293TN), murine Lewis lung carcinoma (LL/2), murine melanoma (B16-F10), murine squamous-cell carcinoma (SCC VII), and murine hepatocellular carcinoma (H22) cell lines were obtained from American Type Cell Culture Collection. The cells were maintained in Dulbecco’s Modified Eagle’s Minimal Medium (DMEM, 4.5 g/L glucose) supplemented with heat-inactivated 10% fetal bovine serum (FBS) in a humidified incubator at 37 °C and 5% CO_2_.

### 4.2. Isolation of Murine Mesenchymal Stem Cells (MSCs) from Bone Marrow

Murine bone marrow MSCs were isolated as follows. Briefly, the exposed bone marrow from the femurs of three mice was washed into a sterile PBS using the 22-gauge needle. Bone marrow cells in 5 mL of PBS were then layered on top of an equal volume of the ice-cold Ficoll-Paque (1.077 g/mL) solution in a 15 mL sterile conical tube and centrifuged at 1900 rpm for 20 min at 4 °C. The mononuclear cell-containing interphase was transferred to a new 15 mL conical tube and washed twice with 10 mL of cold sterile PBS, followed by centrifugation at 1400 rpm for 15 min at 4 °C. The cell pellet was resuspended in pre-warmed DMEM with 20% FBS and plated into 6-well plates with the seeding density of 15 million cells per well. The cells were allowed to adhere for 5 days, after which the media was substituted with DMEM with 10% FBS, which was changed every 2–3 days.

### 4.3. Construction of Plasmids

The plasmids were constructed as previously described [11]. Briefly, the pVSV-dG-GFP plasmid (EH1003, Kerafast, Boston, MA, USA) was used as a vector for generating the pVSV-wtM-GFP and pVSV-dM51-GFP plasmids encoding five viral proteins together with GFP. pVSV-wtM-GFP encodes a wild-type M protein, while pVSV-dM51-GFP encodes an M protein with a deletion of the methionine (ATG) in position 51. pVSV-dM51-mIL12-mGMCSF encodes the mIL12-mGMCSF fusion (2052 bp) in place of the GFP gene. Plasmid pCAG-T7pol, expressing T7 RNA polymerase (T7RNAP) (59926, Addgene, Watertown, MA, USA), was used for the recovery of infectious VSV and as a vector for the construction of helper plasmids expressing VSV-N (pCAG-N), VSV-P (pCAG-P), and VSV-L (pCAG-L) under the control of the CAG promoter. All plasmids were verified by DNA sequencing.

### 4.4. Rescue and Purification of Recombinant VSV

The rescue of rVSV was carried out as previously reported [11]. Briefly, HEK293TN cells were co-transfected with the following plasmids: pCAG-T7pol, pCAG-P, pCAG-L, pCAG-N, pCAG-G, and pVSV-wtM-GFP, pVSV-dM51-GFP, or pVSV-dM51-mIL12-mGMCSF at a 5:5:1:3:4:5 ratio with polyethylenimine (PEI). After 12 h, the media was changed to DMEM, supplemented with 5% FBS, and the cells were cultured at 37 °C in a 5% (*v*/*v*) CO_2_ incubator. The supernatants were collected 96 h later, filtered with a 0.45 μm filter, and used to infect BHK-21 cells. BHK-21 cells, grown overnight in 12-well plates to 70–80% confluency, were infected with 500 μL of rVSV-dM51-GFP, rVSV-wtM-GFP, or rVSV-dM51-mIL12-mGMCSF from the rescue step. One and a half hours later, 500 μL of fresh DMEM (containing 2% FBS and 2 mM L-glutamine) was added. GFP expression was detected by inverted fluorescence microscopy (Carl Zeiss Microscopy GmbH, Jena, Germany) 24 h later. The supernatants were collected after 72 h, centrifuged at 3000× *g* for 10 min at 4 °C, and filtered through a 0.45 μm membrane. For concentration, 4 mL of a 20% sucrose cushion was overlaid onto the virus supernatant. After centrifugation at 120,000× *g* at 4 °C for 1 h, the supernatants were discarded, and the virus pellets were resuspended in 1 mM Tris-HCl (pH 7.5), 1 mM EDTA, and 10% DMSO. Following a 1 h incubation, the samples were purified and concentrated in a sucrose gradient (25, 45, and 60%). The final viral preparation was resuspended in PBS and stored at 4 °C.

### 4.5. Median Tissue Culture Infectious Dose Assay

The quantity of the infectious virus in supernatants was determined by the TCID50 assay as described previously [49]. Briefly, BHK-21 cells were plated in 96-well plates, and 0.5 log serial dilutions of the virus were added to the wells 24 h later. After 72 h, the cells were examined for the presence of a CPE. Final titers (TCID50/mL) were obtained using the Reed-Muench formula.

### 4.6. Infection of Cell Lines

For flow cytometry, rVSV infection was carried out at a 10^6^ TCID50 dose. 1.5 × 10^5^ cells (per well of a 24-well plate) were infected with rVSV in a final volume of 0.5 mL.

For qPCR, B16-F10 and LL/2 cells were cultured in DMEM (Gibco, NY, USA) with 5% FBS and seeded onto 6-well plates at 1 × 10^6^ cells per well. The cells were infected with rVSV-dM51-GFP at a 10^6^ TCID50 dose and incubated at 37 °C in 5% CO_2_ humidified air. The infected cells and the non-infected controls were collected at 12 h after viral infection.

### 4.7. ELISA Assay

The mGMCSF ELISA Kit (EM0089, FineTest, Wuhan, China) was used for detection of mGMCSF presence in virus-containing supernatants according to the manufacturer’s instructions. Absorbance was measured on a ClarioStar plate reader (BMG Labtech, Ortenberg, Germany) at 450 nm.

### 4.8. Flow Cytometry

Twenty-four h post-infection, the number of dead and GFP-positive (GFP+) cells was analyzed by flow cytometry. Briefly, the cells were trypsinized, washed with 500 μL PBS, and resuspended in 250 μL chilled FACS buffer (1 × PBS, 2% FBS, 1 mM EDTA). Propidium iodide (PI) (1:2000 dilution) was added for the detection of dead cells. The data was recorded on the CytoFLEX B2-R2-V0 flow cytometer (Indianapolis, IN, USA) using CytExpert software v1.2 gating on single live cells. The data analysis was carried out using FlowJo™ v10 software.

### 4.9. RNA Isolation, Reverse Transcription (RT) Reaction, and qPCR Assay for the Analysis of Gene Expression in VSV-Infected Cells

Total RNA was isolated from the VSV-infected cells using “Lira” reagent (Biolabmix, Novosibirsk, Russia) according to the manufacturer’s procedure. The RNA concentration and integrity were measured using a NanoDrop ND-1000 spectrophotometer (NanoDrop Technologies, Wilmington, DE, USA). After, the samples were reverse transcribed using the BioMaster RNAscribe RT Plus (5×) kit (Biolabmix, Novosibirsk Russia) according to the manufacturer’s procedure. The cDNA was stored at −20 °C until further use.

The target mRNA sequences of *IFIT1*, *RIG-I*, *N-cadherin*, *p53*, and *IFN-β1* genes, used for primer design, were obtained from the GenBank database (http://www.ncbi.nlm.nih.gov/genbank/, accessed on 13 January 2025). Primers for the amplification of about 100–300 bp gene targets were designed using Primer 3 software (Table S1) and had annealing temperatures of 65 ± 1 °C. The obtained data was normalized to the *GAPDH* gene.

### 4.10. In Vivo Studies

Male C57Bl/6 mice (*n* = 13) with a maximum age of 12 weeks were purchased from the SPF Animal Facility of the Institute of Cytology and Genetics, SB RAS (Novosibirsk, Russia). All animals were housed in standard polypropylene cages at a controlled temperature (25 ± 2 °C), light (12 h of light and dark cycle), and relative humidity (65 ± 5%) conditions and were provided food and water ad libitum. Approximately 5 × 10^6^ LL/2 cells in 200 μL PBS were subcutaneously injected into the left flank. Tumor sizes were measured every 2 days with a caliper, and the volume was calculated using the formula length × width × height × 0.52. When the mean tumor volume reached ~400 mm^3^, the mice received two doses of rVSV-dM51-mIL12-mGMCSF or placebo control intratumorally. Tumor sizes were measured every 2 days. The TGII was calculated using the formula (Vc − Vt)/Vc × 100, where Vc is the average volume of untreated tumors and Vt is the average volume of treated tumors. Moribund mice were sacrificed by cervical dislocation.

### 4.11. Phenotyping of the Spleen Immune Cells

The mice (*n* = 2) that received the rVSV-dM51-mIL12-mGMCSF treatment or PBS were sacrificed. Their spleens were collected and macerated in a 70 μm cell strainer coupled to a 50 mL conical tube filled with PBS. The cell suspension was centrifuged at 1600 rpm for 5 min at 4 °C. The cells were resuspended in 1 mL PBS, and the cell viability was assessed by trypan blue staining. After obtaining single-cell suspension, 1 × 10^6^ of the resulting cells were stained with the anti-mouse antibodies Alexa Fluor 700 anti-CD3 (100216), PerCP/Cyanine 5.5 anti-CD4 (100540), APC anti-CD8a (155006), Brilliant Violet 421 anti-FOXP3 (126419) (all from BioLegend, San Diego, CA, USA), and PE anti-CD25 (E-AB-F1102D, Elabscience, Wuhan, China). The stained cells were acquired on the BD LSR Fortessa cytofluorimeter (Beckman Dickinson, Franklin Lakes, NJ, USA), and data were analyzed using FlowJo™ v10 software.

### 4.12. Statistical Analysis

The statistical analysis of the flow cytometry data from in vitro tests was carried out using an ordinary one-way ANOVA test in GraphPad Prism 8.2.1 software. Results are presented as the mean ± standard deviation of 2–3 biological replicates with a confidence interval. The level of difference is significant when (*) *p*-value < 0.05, (**)—*p*-value < 0.01, (***) *p*-value < 0.001, and (****) *p*-value < 0.0001, and not significant (ns) when *p* > 0.05.

The statistical analysis of the qPCR data was carried out using an unpaired *t*-test in GraphPad Prism 8.2.1 software. The level of difference is significant when (*) *p*-value < 0.05, (**)—*p*-value < 0.01, (***) *p*-value < 0.001, and (****) *p*-value < 0.0001, and not significant (ns) when *p* > 0.05.

The statistical analysis of the flow cytometry data from the in vivo experiment was carried out using an unpaired *t*-test in GraphPad Prism 8.2.1 software.

The statistical analysis for in vivo experiments was performed using Statistica 10.0 (Statsoft, Tulsa, OK, USA), and the graphical visualization was generated using SigmaPlot 12.5 (Systat Software Inc., San Jose, CA, USA). Values are given as mean ± SE; statistical significance was determined using a non-parametric Mann–Whitney U-test.

## Data Availability

The data presented in this study are contained within the article and available upon request from the corresponding author.

## Supplementary Materials

The following supporting information can be downloaded at: https://www.mdpi.com/article/10.3390/ijms26178567/s1.

## Abbreviations

The following abbreviations are used in this manuscript:

OVs	Oncolytic viruses
VSV	Vesicular stomatitis virus
dM51	The methionine deletion in position 51 of M protein
GFP	Green fluorescence protein
mIL12	Mouse interleukin-12
mGMCSF	Mouse granulocyte-macrophage colony-stimulating factor
MSCs	Mesenchymal stem cells
qPCR	Quantitative polymerase chain reaction
TGII	Tumor growth inhibition index
IFNs	Interferons
TME	Tumor microenvironment
GSDME	Gasdermin E
rVSV	Recombinant VSV
wt	Wild-type
LL/2	Lewis lung carcinoma
SCC VII	Squamous-cell carcinoma
H22	Hepatocellular carcinoma
CAG	Cytomegalovirus immediate-early enhancer/chicken β-actin promoter
CPE	Cytopathic effect
NC	Negative control
PBS	Phosphate-buffered saline
Treg	Regulatory T lymphocytes
RFP	Red fluorescence protein
FlaB	Bacterial flagellin (subunit B) of *Vibrio vulnificus*
LDLR	Low-density lipoprotein receptor
EGFR	Epidermal growth factor receptor
RTK	Receptor tyrosine kinase
ERK1/2	Extracellular signal-regulated kinase 1/2
Th1	T-helper 1 lymphocytes
DCs	Dendritic cells
DMEM	Dulbecco’s Modified Eagle’s Minimal Medium
FBS	Fetal bovine serum
T7RNAP	T7 RNA polymerase
PEI	Polyethylenimine
PI	Propidium iodide
RT	Real time

## References

[1] Araghi M., Mannani R., Heidarnejad maleki A., Hamidi A., Rostami S., Safa S.H., Faramarzi F., Khorasani S., Alimohammadi M., Tahmasebi S. (2023). Recent Advances in Non-Small Cell Lung Cancer Targeted Therapy; an Update Review. Cancer Cell Int..

[2] Toropko M., Chuvpilo S., Karabelsky A. (2024). MiRNA-Mediated Mechanisms in the Generation of Effective and Safe Oncolytic Viruses. Pharmaceutics.

[3] Malogolovkin A., Gasanov N., Egorov A., Weener M., Ivanov R., Karabelsky A. (2021). Combinatorial Approaches for Cancer Treatment Using Oncolytic Viruses: Projecting the Perspectives through Clinical Trials Outcomes. Viruses.

[4] Zinovieva M., Ryapolova A., Karabelsky A., Minskaia E. (2024). Oncolytic Vesicular Stomatitis Virus: Optimisation Strategies for Anti-Cancer Therapies. Front. Biosci. Landmark.

[5] Vorona K.A., Moroz V.D., Gasanov N.B., Karabelsky A.V. (2024). Recombinant VSVS: A Promising Tool for Virotherapy. Acta Naturae.

[6] Felt S.A., Grdzelishvili V.Z. (2017). Ecent Advances in Vesicular Stomatitis Virus-Based Oncolytic Virotherapy: A 5-Year Update. J. Gen. Virol..

[7] Connor J.H., Naczki C., Koumenis C., Lyles D.S. (2004). Replication and Cytopathic Effect of Oncolytic Vesicular Stomatitis Virus in Hypoxic Tumor Cells In Vitro and In Vivo. J. Virol..

[8] Zhou Y., Wen F., Zhang P., Tang R., Li Q. (2016). Vesicular Stomatitis Virus Is a Potent Agent for the Treatment of Malignant Ascites. Oncol. Rep..

[9] Zhang Z., Zhang Y., Xia S., Kong Q., Li S., Liu X., Junqueira C., Meza-Sosa K.F., Mok T.M.Y., Ansara J. (2020). Gasdermin E Suppresses Tumour Growth by Activating Anti-Tumour Immunity. Nature.

[10] Lin J., Liu F., Gao F., Chen Y., Wang R., Wang X., Li Y., Li Q., Sun S., Li Z. (2022). Vesicular Stomatitis Virus Sensitizes Immunologically Cold Tumors to Checkpoint Blockade by Inducing Pyroptosis. Biochim. Biophys. Acta Mol. Basis Dis..

[11] Ryapolova A., Minskaia E., Gasanov N., Moroz V., Krapivin B., Egorov A.D., Laktyushkin V., Zhuravleva S., Nagornych M., Subcheva E. (2024). Development of Recombinant Oncolytic RVSV-DM51-MIL12-MGMCSF for Cancer Immunotherapy. Int. J. Mol. Sci..

[12] Marquis K.A., Becker R.L., Weiss A.N., Morris M.C., Ferran M.C. (2020). The VSV Matrix Protein Inhibits NF-ΚB and the Interferon Response Independently in Mouse L929 Cells. Virology.

[13] Hastie E., Grdzelishvili V.Z. (2012). Vesicular Stomatitis Virus as a Flexible Platform for Oncolytic Virotherapy against Cancer. J. Gen. Virol..

[14] Goad D.W., Nesmelova A.Y., Yohe L.R., Grdzelishvili V.Z. (2023). Intertumoral Heterogeneity Impacts Oncolytic Vesicular Stomatitis Virus Efficacy in Mouse Pancreatic Cancer Cells. J. Virol..

[15] Shakiba Y., Vorobyev P.O., Naumenko V.A., Kochetkov D.V., Zajtseva K.V., Valikhov M.P., Yusubalieva G.M., Gumennaya Y.D., Emelyanov E.A., Semkina A.S. (2023). Oncolytic Efficacy of a Recombinant Vaccinia Virus Strain Expressing Bacterial Flagellin in Solid Tumor Models. Viruses.

[16] Mayer S., Milo T., Isaacson A., Halperin C., Miyara S., Stein Y., Lior C., Pevsner-Fischer M., Tzahor E., Mayo A. (2023). The Tumor Microenvironment Shows a Hierarchy of Cell-Cell Interactions Dominated by Fibroblasts. Nat. Commun..

[17] de Visser K.E., Joyce J.A. (2023). The Evolving Tumor Microenvironment: From Cancer Initiation to Metastatic Outgrowth. Cancer Cell.

[18] Anderson N.M., Simon M.C. (2020). The Tumor Microenvironment. Curr. Biol..

[19] Xu C., Lin L., Cao G., Chen Q., Shou P., Huang Y., Han Y., Wang Y., Shi Y. (2014). Interferon-α-Secreting Mesenchymal Stem Cells Exert Potent Antitumor Effect in Vivo. Oncogene.

[20] Shou P., Chen Q., Jiang J., Xu C., Zhang J., Zheng C., Jiang M., Velletri T., Cao W., Huang Y. (2016). Type i Interferons Exert Anti-Tumor Effect via Reversing Immunosuppression Mediated by Mesenchymal Stromal Cells. Oncogene.

[21] Melzer M.K., Lopez-Martinez A., Altomonte J. (2017). Oncolytic Vesicular Stomatitis Virus as a Viro-Immunotherapy: Defeating Cancer with a “Hammer” and “Anvil”. Biomedicines.

[22] Finkelshtein D., Werman A., Novick D., Barak S., Rubinstein M. (2013). LDL Receptor and Its Family Members Serve as the Cellular Receptors for Vesicular Stomatitis Virus. Proc. Natl. Acad. Sci. USA.

[23] Yang C., Gagnon C., Hou X., Hardy P. (2010). Low Density Lipoprotein Receptor Mediates Anti-VEGF Effect of Lymphocyte T-Derived Microparticles in Lewis Lung Carcinoma Cells. Cancer Biol. Ther..

[24] Gal D., Macdonald P.C., Porter J.C., Smith J.W., Simpson E.R. (1981). Effect of Cell Density and Confluency on Cholesterol Metabolism in Cancer Cells in Monolayer Culture1. Cancer Res..

[25] Kelishadi R., Hashemipour M., Sarrafzadegan N., Mohammadifard N., Alikhasy H., Beizaei M., Sajjadi F., Poursafa P., Amin Z., Ghatreh-Samani S. (2010). Effects of a Lifestyle Modification Trial among Phenotypically Obese Metabolically Normal and Phenotypically Obese Metabolically Abnormal Adolescents in Comparison with Phenotypically Normal Metabolically Obese Adolescents. Matern. Child Nutr..

[26] Ayala-Breton C., Barber G.N., Russell S.J., Peng K.-W. (2011). Retargeting Vesicular Stomatitis Virus Using Measles Virus Envelope Glycoproteins. Hum. Gene Ther..

[27] Hu W., Zhang S., Shen Y., Yang Q. (2018). Epidermal Growth Factor Receptor Is a Co-Factor for Transmissible Gastroenteritis Virus Entry. Virology.

[28] Chen J., Zeng F., Forrester S.J., Eguchi S., Zhang M.-Z., Harris R.C. (2016). Expression and Function of the Epidermal Growth Factor Receptor in Physiology and Disease. Physiol. Rev..

[29] Li T., Zhang C., Zhao G., Zhang X., Hao M., Hassan S., Zhang M., Zheng H., Yang D., Liu L. (2020). IGFBP2 Regulates PD-L1 Expression by Activating the EGFR-STAT3 Signaling Pathway in Malignant Melanoma. Cancer Lett..

[30] Boone B., Jacobs K., Ferdinande L., Taildeman J., Lambert J., Peeters M., Bracke M., Pauwels P., Brochez L. (2011). EGFR in Melanoma: Clinical Significance and Potential Therapeutic Target. J. Cutan. Pathol..

[31] Gross A., Niemetz-Rahn A., Nonnenmacher A., Tucholski J., Keilholz U., Fusi A. (2015). Expression and Activity of EGFR in Human Cutaneous Melanoma Cell Lines and Influence of Vemurafenib on the EGFR Pathway. Target. Oncol..

[32] Selman M., Rousso C., Bergeron A., Son H.H., Krishnan R., El-Sayes N.A., Varette O., Chen A., Le Boeuf F., Tzelepis F. (2018). Multi-Modal Potentiation of Oncolytic Virotherapy by Vanadium Compounds. Mol. Ther..

[33] Bergeron A., Kostenkova K., Selman M., Murakami H.A., Owens E., Haribabu N., Arulanandam R., Diallo J.-S., Crans D.C. (2019). Enhancement of Oncolytic Virotherapy by Vanadium(V) Dipicolinates. BioMetals.

[34] Her L.-S., Lund E., Dahlberg J.E. (1997). Inhibition of Ran Guanosine Triphosphatase-Dependent Nuclear Transport by the Matrix Protein of Vesicular Stomatitis Virus. Science.

[35] Von Kobbe C., Van Deursen J.M.A., Rodrigues J.P., Sitterlin D., Bachi A., Wu X., Wilm M., Carmo-Fonseca M., Izaurralde E. (2000). Vesicular Stomatitis Virus Matrix Protein Inhibits Host Cell Gene Expression by Targeting the Nucleoporin Nup98. Mol. Cell.

[36] Terstegen L., Gatsios P., Ludwig S., Pleschka S., Jahnen-Dechent W., Heinrich P.C., Graeve L. (2001). The Vesicular Stomatitis Virus Matrix Protein Inhibits Glycoprotein 130-Dependent STAT Activation. J. Immunol..

[37] Lai M.D., Yen M.C., Lin C.M., Tu C.F., Wang C.C., Lin P.S., Yang H.J., Lin C.C. (2009). The Effects of DNA Formulation and Administration Route on Cancer Therapeutic Efficacy with Xenogenic EGFR DNA Vaccine in a Lung Cancer Animal Model. Genet. Vaccines Ther..

[38] Piao Y., Jiang H., Alemany R., Krasnykh V., Marini F.C., Xu J., Alonso M.M., Conrad C.A., Aldape K.D., Gomez-Manzano C. (2009). Oncolytic Adenovirus Retargeted to Delta-EGFR Induces Selective Antiglioma Activity. Cancer Gene Ther..

[39] Uchida H., Marzulli M., Nakano K., Goins W.F., Chan J., Hong C.S., Mazzacurati L., Yoo J.Y., Haseley A., Nakashima H. (2013). Effective Treatment of an Orthotopic Xenograft Model of Human Glioblastoma Using an EGFR-Retargeted Oncolytic Herpes Simplex Virus. Mol. Ther..

[40] Yang L., Xu J., Guo L., Guo T., Zhang L., Feng L., Chen H., Wang Y. (2018). Porcine Epidemic Diarrhea Virus-Induced Epidermal Growth Factor Receptor Activation Impairs the Antiviral Activity of Type I Interferon. J. Virol..

[41] Mitchell H.D., Eisfeld A.J., Stratton K.G., Heller N.C., Bramer L.M., Wen J., McDermott J.E., Gralinski L.E., Sims A.C., Le M.Q. (2019). The Role of EGFR in Influenza Pathogenicity: Multiple Network-Based Approaches to Identify a Key Regulator of Non-Lethal Infections. Front. Cell Dev. Biol..

[42] Ueki I.F., Min-Oo G., Kalinowski A., Ballon-Landa E., Lanier L.L., Nadel J.A., Koff J.L. (2013). Respiratory Virus–Induced EGFR Activation Suppresses IRF1-Dependent Interferon λ and Antiviral Defense in Airway Epithelium. J. Exp. Med..

[43] Lasek W., Zagożdżon R., Jakobisiak M. (2014). Interleukin 12: Still a Promising Candidate for Tumor Immunotherapy?. Cancer Immunol. Immunother..

[44] Wang G., Kang X., Chen K.S., Jehng T., Jones L., Chen J., Huang X.F., Chen S.Y. (2020). An Engineered Oncolytic Virus Expressing PD-L1 Inhibitors Activates Tumor Neoantigen-Specific T Cell Responses. Nat. Commun..

[45] Wang R., Chen J., Wang W., Zhao Z., Wang H., Liu S., Li F., Wan Y., Yin J., Wang R. (2022). CD40L-Armed Oncolytic Herpes Simplex Virus Suppresses Pancreatic Ductal Adenocarcinoma by Facilitating the Tumor Microenvironment Favorable to Cytotoxic T Cell Response in the Syngeneic Mouse Model. J. Immunother. Cancer.

[46] Nakao S., Arai Y., Tasaki M., Yamashita M., Murakami R., Kawase T., Amino N., Nakatake M., Kurosaki H., Mori M. (2020). Intratumoral expression of IL-7 and IL-12 Using an Oncolytic Virus Increases Systemic Sensitivity to Immune Checkpoint Blockade. Sci. Transl. Med..

[47] Dougan M., Dranoff G., Dougan S.K. (2019). GM-CSF, IL-3, and IL-5 Family of Cytokines: Regulators of Inflammation. Immunity.

[48] Vasileva N., Ageenko A., Dmitrieva M., Nushtaeva A., Mishinov S., Kochneva G., Richter V., Kuligina E. (2021). Double Recombinant Vaccinia Virus: A Candidate Drug against Human Glioblastoma. Life.

[49] Lei C., Yang J., Hu J., Sun X. (2021). On the Calculation of TCID50 for Quantitation of Virus Infectivity. Virol. Sin..

