# Supplementary material for: Recombinant Oncolytic Vesicular Stomatitis Virus Expressing Mouse Interleukin-12 and Granulocyte-Macrophage Colony-Stimulating Factor (rVSV-dM51-mIL12-mGMCSF) for Immunotherapy of Lung Carcinoma
Source: Int J Mol Sci. 2025 Sep 3;26(17):8567. doi: 10.3390/ijms26178567 (PMC12429742; doi:10.3390/ijms26178567)
Supplement: Supplementary file 1 [file ijms-26-08567-s001.zip › Figure S1.pdf]

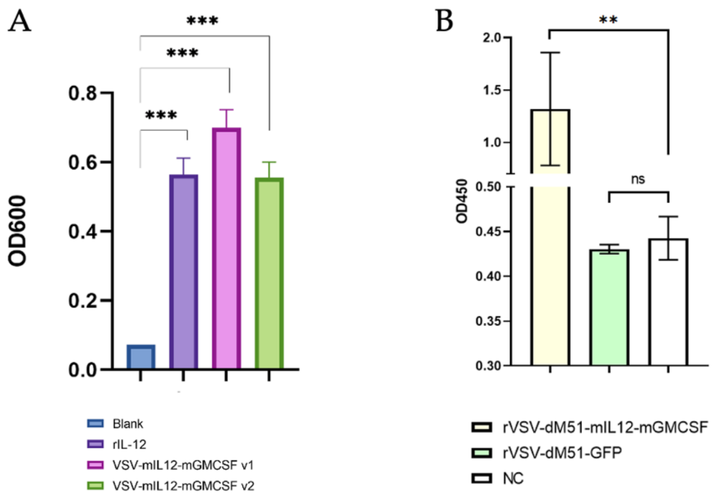

Figure S1. Assessment of mIL-12 activity in HEK-Blue IL-12 cells and mGMCSF presence by ELISA analysis. **A**. Assessment of IL-12 activity in HEK-Blue IL-12 cells IL-12 activity in supernatants containing rVSV-mIL12-mGMCSF amplified in two different volumes, T25 (v1) and T175 (v2) flasks, was assessed in HEK-Blue IL-12 cells by quantifying the expression levels of SEAP. SEAP expression levels were compared to the positive control, recombinant IL-12 (rIL-12), used at 10 ng/mL. Absorbance was measured on a ClarioStar plate reader at 650 nm. The statistical analysis was carried out using ordinary one-way ANOVA and Sidak's multiple comparison test in GraphPad Prism (*** $p \leq 0.001$). **B**. ELISA of mGMCSF in supernatants containing rVSV-dM51-mIL12-mGMCSF and rVSV-dM51-GFP was compared to the negative control. Absorbance was measured on a ClarioStar plate reader at 450 nm. The statistical analysis was carried out using ordinary one-way ANOVA test in GraphPad Prism (** p-val < 0.01, not significant (ns)– p-val > 0.05).
