# Supplementary material for: Recombinant Oncolytic Vesicular Stomatitis Virus Expressing Mouse Interleukin-12 and Granulocyte-Macrophage Colony-Stimulating Factor (rVSV-dM51-mIL12-mGMCSF) for Immunotherapy of Lung Carcinoma
Source: Int J Mol Sci. 2025 Sep 3;26(17):8567. doi: 10.3390/ijms26178567 (PMC12429742; doi:10.3390/ijms26178567)
Supplement: Supplementary file 1 [file ijms-26-08567-s001.zip › IJMS Table S1.pdf]

Cell line	VSV	% GFP+ cells				Average	M	
B16-F10 24 hrs	dM51 GFP	3	2	9	9	6	1452	1499
	wtM GFP	11	16	24	27	20	1959	1858
B16-F10 48 hrs	dM51 GFP	10	9	1	1	5	1365	1456
	wtM GFP	14	9	21	20	16	2018	2001
LL/2 24 hrs	dM51 GFP	60	66	58	64	62	8484	6459
	wtM GFP	74	77	66	67	71	104227	106044
LL/2 48 hrs	dM51 GFP	24	26	20	19	22	2384	2443
	wtM GFP	20	21	25	27	23	3496	2888
SCC VII 24 hrs	dM51 GFP	23	22	25	24	24	1949	1923
	wtM GFP	2	3	7	8	5	1697	1734
SCC VII 48 hrs	dM51 GFP	29	26	23	22	25	2371	2470
	wtM GFP	60	64	51	55	58	7676	4339

FI		MFI Average	% PI+ cells				Average
1064	1100	1279	23	19	24	24	23
1337	1220	1594	37	40	46	40	41
1757	1780	1590	42	40	33	28	36
2129	2083	2058	96	97	94	95	96
11849	7748	8635	69	65	64	58	64
80176	81570	93004	97	96	99	99	98
2594	2423	2461	82	79	76	77	79
3576	2841	3200	100	100	99	100	100
2483	2538	2223	25	21	18	16	20
1281	1204	1479	19	16	8	6	12
2333	2545	2430	67	66	71	64	67
19225	14583	11456	79	78	88	51	74
