# Supplementary material for: Recombinant Oncolytic Vesicular Stomatitis Virus Expressing Mouse Interleukin-12 and Granulocyte-Macrophage Colony-Stimulating Factor (rVSV-dM51-mIL12-mGMCSF) for Immunotherapy of Lung Carcinoma
Source: Int J Mol Sci. 2025 Sep 3;26(17):8567. doi: 10.3390/ijms26178567 (PMC12429742; doi:10.3390/ijms26178567)
Supplement: Supplementary file 1 [file ijms-26-08567-s001.zip › IJMS Table S2.pdf]

	Dead					
Cell line	VSV	PI+				PI+ Average
B16-F10 24 hrs	mIL12-mGMCSF	17	20	17	17	17
	dM51 GFP	23	19	24	24	23
	NC	10	10	17	21	14
LL/2 24 hrs	mIL12-mGMCSF	16	21	20	22	20
	dM51 GFP	69	65	64	58	64
	NC	6	5	5	9	6
SCC VII 24 hrs	mIL12-mGMCSF	14	12	6	7	10
	dM51 GFP	25	21	18	16	20
	NC	3	10	9	3	6
H22 24 hrs	mIL12-mGMCSF	84	83	85	84	84
	dM51 GFP	96	95	96	95	96
	NC	6	6	5	6	6
MSC 24 hrs	mIL12-mGMCSF	42	43	41	41	42
	dM51 GFP	99	99	99	99	99
	NC	9	12	7	9	9
