# Supplementary material for: Recombinant Oncolytic Vesicular Stomatitis Virus Expressing Mouse Interleukin-12 and Granulocyte-Macrophage Colony-Stimulating Factor (rVSV-dM51-mIL12-mGMCSF) for Immunotherapy of Lung Carcinoma
Source: Int J Mol Sci. 2025 Sep 3;26(17):8567. doi: 10.3390/ijms26178567 (PMC12429742; doi:10.3390/ijms26178567)
Supplement: Supplementary file 1 [file ijms-26-08567-s001.zip › IJMS Table S3.pdf]

Cell popullation	Treatment			Average
CD3+	rVSV-dM51-mIL12-mGMCSF	10	9	10
	NC	14	10	12
CD3+ CD4+	rVSV-dM51-mIL12-mGMCSF	40	40	40
	NC	41	42	41
CD3+ CD8+	rVSV-dM51-mIL12-mGMCSF	30	35	32
	NC	45	38	42
CD3+ CD4+ FoxP3+	rVSV-dM51-mIL12-mGMCSF	2	3	3
	NC	4	3	4
