# Supplementary material for: Recombinant Oncolytic Vesicular Stomatitis Virus Expressing Mouse Interleukin-12 and Granulocyte-Macrophage Colony-Stimulating Factor (rVSV-dM51-mIL12-mGMCSF) for Immunotherapy of Lung Carcinoma
Source: Int J Mol Sci. 2025 Sep 3;26(17):8567. doi: 10.3390/ijms26178567 (PMC12429742; doi:10.3390/ijms26178567)
Supplement: Supplementary file 1 [file ijms-26-08567-s001.zip › IJMS Table S4.pdf]

Gene	Sequence (5' → 3')
mIFIT1_f	TACAGGCTGGAGTGTGCTGAGA
mIFIT1_r	CTCCACTTTCAGAGCCTTCGCA
mRIGI_f	AGCCAAGGATGTCTCCGAGGAA
mRIGI_r	ACACTGAGCACGCTTTGTGGAC
mN-cad_f	TGAAACGGCGGGATAAAGAG
mN-cad_r	GGCTCCACAGTATCTGGTTG
mp53_f	CACAGCACATGACGGAGGTC
mp53_r	TCCTTCCACCCGGATAAGATG
mIL-1b_f	TGGACCTTCCAGGATGAGGACA
mIL-1b_r	GTTCATCTCGGAGCCTGTAGTG
mIFNb1_f	CAGCACTGGGTGGAATGAGA
mIFNb1_r	GTGGAGAGCAGTTGAGGACA
mGAPDH_f	CATCACTGCCACCCAGAAGACTG
mGAPDH_r	ATGCCAGTGAGCTTCCCGTTCAG

Supplementary Table S4. qPCR primers used in this study.
